# High response rates with single-agent belantamab mafodotin in relapsed systemic AL amyloidosis

**DOI:** 10.1038/s41408-022-00717-2

**Published:** 2022-09-05

**Authors:** Jahanzaib Khwaja, Joshua Bomsztyk, Shameem Mahmood, Brendan Wisniowski, Raakhee Shah, Anish Tailor, Kwee Yong, Rakesh Popat, Neil Rabin, Charalampia Kyriakou, Jonathan Sive, Simona Degli Esposti, Daniel F. P. Larkin, Sarah Worthington, Alyse Hart, Emma Dowling, Nuno Correia, Ceri Bygrave, Andrzej Rydzewski, Krzysztof Jamroziak, Ashutosh D. Wechalekar

**Affiliations:** 1 Department of Haematology, University College London Hospital, London, UK; 2 National Amyloidosis Centre, University College London (Royal Free Campus), London, UK; 3 Moorfields Eye Hospital NHS Foundation Trust and UCL Institute of Ophthalmology, London, UK; 4 Department of Haematology, University Hospital of Wales, Cardiff, UK; 5 Department of Internal Medicine, Nephrology and Transplantation Medicine, Central Clinical Hospital of the Ministry of Internal Affairs, Warsaw, Poland; 6 Department of Hematology, Transplantation and Internal Medicine, Medical University of Warsaw, Warsaw, Poland

**Keywords:** Myeloma, Cancer immunotherapy

Dear editor,

Systemic amyloid light-chain (AL) amyloidosis is a rare plasma cell disorder caused by the extracellular deposition of misfolded immunoglobulin light chains as protein fibrils in tissues. This leads to vital organ damage. It is incurable and has a relapsing–remitting course. With a lack of approved treatments at relapse, therapies for relapsed multiple myeloma are used in AL amyloidosis. However, this may be challenging due to underlying organ dysfunction, most commonly cardiac and renal impairment. Most data are from the use of immunomodulatory agents and, lately, daratumumab. Options for patients who relapse after daratumumab in AL amyloidosis remain unclear.

Belantamab mafodotin is an antibody–drug conjugate linked to monomethyl auristatin F, which targets B-cell maturation antigen and has been approved for relapsed refractory myeloma. In patients with multiply pre-treated myeloma, the pivotal DREAMM-2 phase II trial reported on 96 patients after greater than three prior lines of therapy showing an overall response rate (ORR) of 32% as a single-agent intravenous dose of 2.5 mg/kg administered every 3 weeks [1].

A retrospective case series of six patients with relapsed AL amyloidosis with myeloma [2] recently reported on outcomes in a cohort of predominantly cardiac AL amyloidosis (5/6 patients) with a complete response (CR) rate of 50%. A phase II trial is recruiting patients with refractory amyloidosis (NCT04617925) but excludes those with renal impairment (estimated glomerular filtration rate (eGFR) <30 ml/min). Renal involvement causing chronic kidney disease (CKD) is present in 70% of patients with systemic AL at diagnosis. Renal amyloidosis often progresses to end-stage renal failure [3] and these patients are almost universally excluded from clinical trials.

We report our results using belantamab mafodotin monotherapy for the treatment of patients with relapsed refractory AL amyloidosis including those with CKD.

## Methods

This series includes all patients treated with belantamab mafodotin monotherapy at a standard dose (2.5 mg/kg) or dose reduction at the United Kingdom National Amyloidosis Centre between April 2021 and February 2022. All patients underwent standardised assessments including measurement of cardiac biomarkers, clonal parameters and imaging as appropriate. Organ involvement, haematological and organ response assessment was reported as per International Society of Amyloidosis consensus criteria [4]. All patients had ophthalmology assessment in advance of treatment initiation and during therapy in keeping with the licence. Corneal abnormality was graded in accordance with the report by Farooq et al. [5]. Other adverse events were graded in accordance with National Cancer Institute Common Terminology Criteria for Adverse Events version 5.0.

## Results and discussion

Eleven patients were included (eight male, three female). Baseline characteristics and responses are illustrated in Table 1. The median age at belantamab mafodotin initiation was 65 years (range 42–74) and 3 (27%) patients were aged over 70 years. Eight patients (73%) had λ AL-type and 3 (27%) κ AL-type. At diagnosis, the median involved free light-chain concentration was 534 (range 73–7181) mg/l. A median of two organs was involved at baseline (range 1–3): 9 (82%) had renal involvement and 4 (36%) had cardiac involvement. The median eGFR at the time of first belantamab mafodotin dose was 43 ml/min (range 7–120).

**Table 1:** Baseline characteristics and responses.

Patient number	1	2	3	4	5	6	7	8	9	10	11
Age, gender	60, F	72, M	42, F	65, F	64, M	74, M	63, M	73, M	66, M	67, M	58, M
iFLC at diagnosis, mg/l	2368	97	1069	73	499	7181	2330	1940	534	495	235
Organ involvement											
Cardiac	✓	x	✓	x	✓	x	✓	x	x	x	x
Renal	✓	✓	✓	✓	x	✓	✓	x	✓	✓	✓
Liver	x	✓	x	✓	x	x	x	x	x	✓	x
Soft tissue	✓	✓	x	x	✓	x	x	✓	✓	x	x
Prior lines of therapy	4	2	3	4	3	3	4	4	3	5	3
Prior therapy											
Immunomodulatory drug	✓	x	✓	✓	✓	✓	✓	✓	✓	✓	✓
Proteasome inhibitor	✓	✓	✓	✓	✓	✓	✓	✓	✓	✓	✓
Anti-CD38	✓	✓	✓	✓	✓	x	✓	✓	x	✓	✓
Melphalan ASCT	x	x	x	x	x	x	✓	✓	✓	✓	x
iFLC at belantamab mafodotin initiation, mg/l	267	31	194	172	57	341	109	453	89	133	45
Doses delivered	11	4	6	4	6	6	8	1	7	6	4
Dose reduction	✓	✓	x	✓	✓	x	✓	x	✓	x	✓
Toxicity											
Keratopathy, grade	1	1	2	—	3	—	2	3	2	1	—
Dyspnoea, grade	—	1	—	—	—	—	—	—	—	—	—
Liver dysfunction, grade	—	1	—	—	—	—	—	—	—	—	—

*iFLC* involved free light chain, *ASCT* autologous stem cell transplant.

The median time from AL amyloidosis diagnosis to first administration of belantamab mafodotin was 58 (range 12–154) months (~4.8 years) with a median of three prior lines of treatment (range 2–5). Prior therapies included immunomodulatory drugs (91%), proteasome inhibitors (100%) and anti-CD38 antibody (82%) treatment. Four patients (36%) had undergone prior melphalan-conditioned autologous stem cell transplantation.

**Fig. 1:**
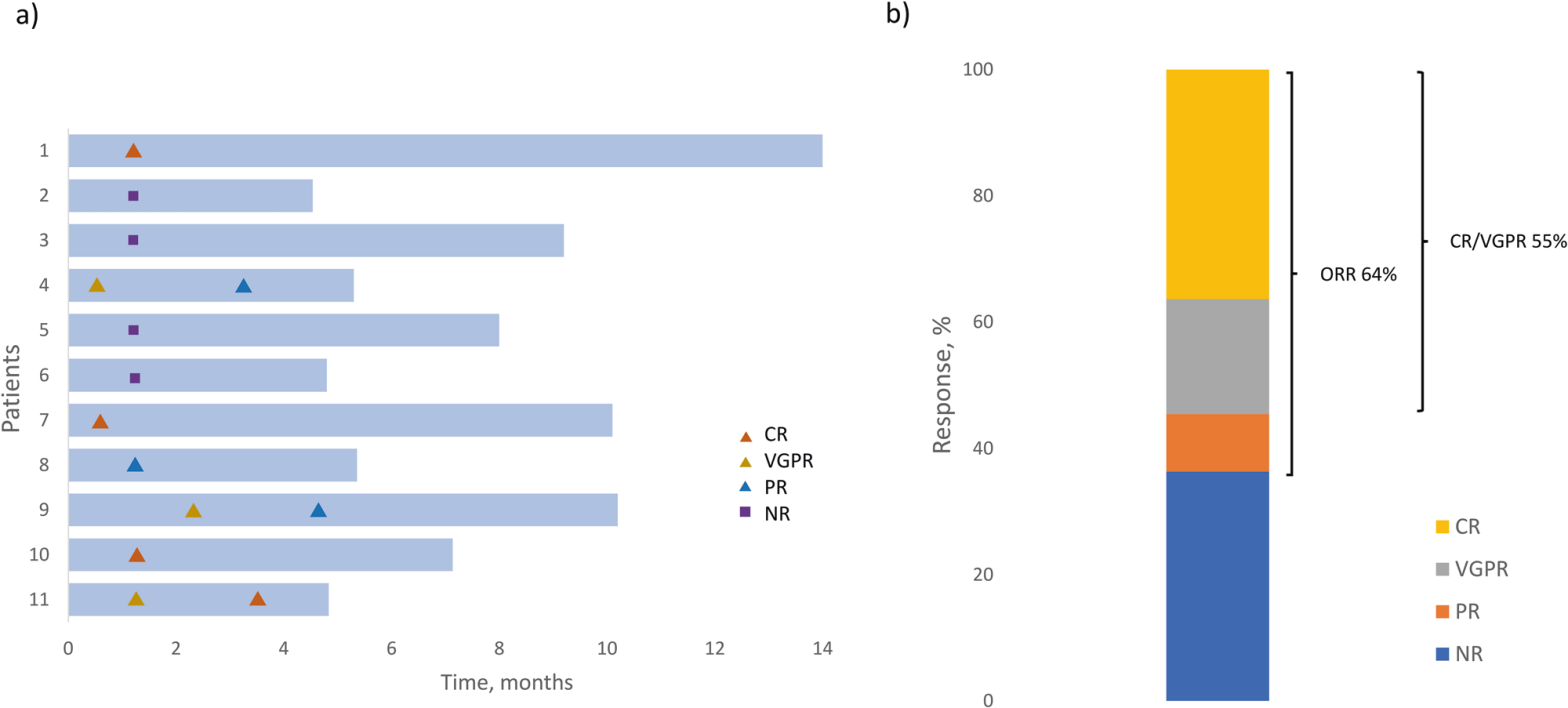
Treatment response rates. **a** Overall response rates; **b** Time to treatment response; Key: CR complete response, VGPR very good partial response, NR no response, ORR overall response rate.

At data cut-off, patients have received a median of 6 (range 1–11) doses of belantamab mafodotin. Response rates are shown in Fig. 1. Eight patients (73%) are still on therapy, ORR (partial response [PR] or better) was 64%. CR or very good partial response (VGPR) was achieved in 6 patients (55%). Reasons for treatment discontinuation (*n* = 1 each, 27% overall) were progressive disease and non-response or toxicity, respectively. At data cut-off, all patients were alive. At a median follow-up of 7.1 months (range 4.5–14.0), progression-free survival was 83% (95% confidence interval 27–97) and the median progression-free survival was not reached.

The most frequent adverse event was keratopathy, in all patients a bilateral microcystic corneal epitheliopathy. This occurred in 8 (73%) patients (grade 1–2: 55%; grade 3: 18%), necessitating dose and schedule modification of the three-weekly delivery in 4 (36%) patients. Ocular adverse events improved after treatment delay (increasing drug intervals to 4–6 weekly) and topical emollients with/without topical corticosteroids. One patient required treatment cessation due to ocular toxicity preventing further dose administration despite achieving PR after just one dose (patient 8).

One patient developed transient grade 1 dyspnoea and asymptomatic liver dysfunction which is similar to the rate reported in DREAMM-2. In our cohort, no patients developed cytopenia, which differed from DREAMM-2, which reported thrombocytopenia in 35% and anaemia in 24% as the most common adverse events after keratopathy. The only other series of reported belantamab mafodotin use in AL amyloidosis described thrombocytopenia in 17% (1/6) [2]. In our cohort, no infusion reactions were reported nor infections observed beyond COVID (two patients had mild infections not requiring hospital admission).

The majority of the cohort required dose reduction either at initiation (patient 4, due to end-stage renal failure and haemodialysis; patient 11, post-renal transplant) or during therapy (5/11; 45%: 3–1.9 mg/kg, 2–1.25 mg/kg). Only one patient remained on the standard dose of 2.5 mg/kg for ≥3 cycles. Four patients had an eGFR <30 ml/min with one patient experiencing grade 1 keratopathy. Two patients (patients 4 and 11) with end-stage renal failure commenced a dose of 1.25 mg/kg and achieved a VGPR and CR, respectively, with no additional toxicity. Patient 11 was treated with belantamab mafodotin after renal transplantation and was taking tacrolimus and mycophenolate mofetil as immunosuppression—we did not see any significant toxicity with a four-weekly dosing schedule and the patient achieved a CR at cycle 3. Patient 3 had a 42% reduction in the involved serum-free light chain after two doses but then had a prolonged gap due to keratopathy and lost the response. There were no treatment-related deaths, hospitalisations due to belantamab mafodotin and cardiac or renal toxicities observed in our cohort.

Belantamab mafodotin demonstrates significant activity in patients with heavily pre-treated AL amyloidosis with an ORR of 64%. Given the low grade underlying clonal dyscrasia in AL amyloidosis, these response rates appear to compare favourably with trial and real-world data of 30% achieving responses in relapsed myeloma. Effective novel therapies in multiply relapsed refractory AL amyloidosis are welcomed as data in this setting is scant. We recently reported real-world longitudinal data showing good outcomes and that responses do not significantly worsen with subsequent relapses in AL amyloidosis with 40–50% achieving at least a VGPR [6]. In the current cohort, apart from reversible keratopathy requiring dose modification and one treatment cessation, no other substantial toxicity was observed. Crucially, the common problems with AL amyloidosis treatment, often caused by steroids, like fluid retention and fatigue were not seen with belantamab mafodotin. Corneal toxicity was not unexpected; baseline and sequential ophthalmic examinations between belantamab mafodotin treatments allow monitoring for keratopathy. Of the current cohort, five patients would have been trial ineligible for the current prospective phase II trial (four due to renal impairment and one due to cardiac biomarkers). Two patients with severe renal impairment (stage V CKD) and one patient post-renal transplant tolerated treatment without additional toxicity and had good responses.

Our data has inherent limitations due to its retrospective nature and small sample size; however, we demonstrate good efficacy and tolerability of belantamab mafodotin in multiply relapsed AL amyloidosis including efficacy in patients with renal impairment. In summary, Belantamab mafodotin shows efficacy in our series of patients with multiply relapsed AL amyloidosis including those excluded from clinical trials. Further evaluation in prospective trials including those patients with advanced renal and cardiac disease is welcomed.

## Data Availability

The data that support the findings of this study are available on request from the corresponding author. The data are not publicly available due to privacy or ethical restrictions.
